# Undergraduate Research Teams That Build Bridges, Produce Publishable Research, and Strengthen Grant Proposals

**DOI:** 10.3389/fpsyg.2019.00133

**Published:** 2019-02-04

**Authors:** Brian Detweiler-Bedell, Jerusha B. Detweiler-Bedell

**Affiliations:** Department of Psychology, Lewis & Clark College, Portland, OR, United States

**Keywords:** undergraduate research, mentoring, team-based learning, psychological science, diversity and inclusion

## Introduction

Engaging undergraduates in the research process is one of the most rewarding aspects of being a professor because it more deeply connects us to our work and helps shape the professional futures of students by immersing them in the culture of research (including peer-to-peer mentoring and authoring publications; Russell et al., 2007). But there is a real trick to working with undergraduates in a way that both shapes students' futures *and* produces high-quality, publishable research because mentors must invest a great deal of time developing undergraduates' technical and writing abilities, and this effort is time not spent on the research itself. In this article, we describe a powerful, flexible approach that makes the production of publishable research possible.

For context, we teach and conduct research at a small liberal arts college with a population of just over 2,000 undergraduates. Research at primarily undergraduate institutions (PUIs) does not benefit from a system of graduate students, post-docs, and paid research staff, so we have found it necessary to develop a structured, team-based approach to faculty-student research that provides excellent mentorship and produces publishable research (see Detweiler-Bedell and Detweiler-Bedell, 2013). Importantly, this team-based model can be put into practice with a broad array of students, including underrepresented and first-generation students. What we have learned in adopting this approach reflects a deeper appreciation of why certain details of faculty-student research (i.e., systematically laddering students' experiences to foster a sense of belonging and increase the efficiency of research) matter as much as they do, as well as the importance of best practices in designing and managing effective teams. Specifically, the most effective teams, according to Hackman (2002): (1) have clear boundaries, interdependence, and stability of membership (yet are semi-permeable) over time; (2) are given and share a compelling direction; (3) utilize a structure that enables teamwork; (4) have a supportive social context; and (5) receive competent coaching to help navigate challenges and take advantage of opportunities.

Although it is beyond the scope of this short article to describe every aspect of our approach to structuring and mentoring undergraduate research teams, the value of this approach to a few key aspects of producing publishable research stand out: enhancing students' sense of belonging in order to build bridges to more diverse student populations, teaching collaborative writing, and securing funding for one's research.

## Using a Team-Based Approach

In our lab, we organize students into multiple, 3-person laddered teams (Detweiler-Bedell and Detweiler-Bedell, 2007), with an experienced student (the team leader; usually a senior psychology major with past experience in our lab) mentoring a mid-level student (the team associate; usually a sophomore or junior) alongside a student new to the research lab (the team assistant; see Table 1). As the faculty mentors, we give teams a clear, compelling vision and direction for their projects, but the teams work on their own at least twice weekly and have great autonomy over their process of working together. To provide a supportive context for this work (i.e., an iterative system of technical guidance and oversight), we provide 3–4 hours of leadership training and meet regularly with the team leaders to ensure that clear research protocols are developed and followed. Likewise, we meet weekly with the lab as a whole, enabling us to assess each team's progress and provide educational lessons, coaching, and oversight on particular research tasks. We find that undergraduates need insight over simple direction, so we ask them *why* even the smallest technical details might matter to the research project's overall vision, and we guide each of these conversations to a clear principle that informs the work.

**Table 1:** Team structure, recruitment, and responsibilities.

**Role**	**Team assistant**	**Team associate**	**Team leader**
Time commitment	4–6 h/week	6–8 h/week	8–10 h/week
Class standing	First-years & sophomores new to psychology	Advanced sophomore, junior & senior psychology students	Advanced junior or senior psychology students who are veteran lab members
Recruitment strategies	Recruitment of first-year, first-generation students through a college-wide program Identification of students from classes taught by mentors such as Introductory Psychology & Statistics Word of mouth	Retention of team assistants (who become associates in year 2) Identification of students from classes such as Research Methodology and mid-level psychology classes taught by mentors Word of mouth	Selection of students who have at least 1 year of experience in the lab & are prepared to take on this level of commitment Preparation and training of leaders through weekly mentor meetings outside the regular lab meetings
Tasks	Attend weekly lab & team meetings Initial training in how to conduct literature searches, design surveys, & run experiments Read relevant background literature Assist in design of experimental materials Help run experiments Present research to lab Assist with conference presentation preparation	Attend weekly lab & team meetings Mentor team assistant Collect and read relevant background literature Design experimental materials Run experiments Assist with IRB applications & data preparation and analysis Present research to lab Assist with conference presentation preparation	Attend weekly lab, team, supervisory & leadership meetings Mentor assistant & associate; Integrate team members' efforts, providing work one consistent “voice” Organize & oversee daily operations: choice of background literature, experimental design, IRB applications, data collection & analysis Present research to lab Lead conference presentation preparation Assist mentor with manuscript writing

Team leaders then take ownership of the day-to-day operations of the lab, which sets a powerful example for the less experienced students. Leaders are charged with having their team work interdependently and in a manner that transmits the skills necessary for the team associate and assistant to carry out high-quality research. This places significant responsibility on our team leaders, who are hand-selected based on their development as effective near-peer mentors. We rarely encounter problematic dynamics that stem from our student leaders, in part because only about one-third of our students ultimately grow into this role. Moreover, the lab is a close-knit environment, and even small issues are noticed quickly because we hold each other accountable to the principles and practices we introduce during leadership training.

We make clear to potential lab members at the time of their interview that most students stay with the lab over a number of semesters (and even years) and that we hope, if the fit is right, they will too. Team membership does remain relatively stable over time, with most students engaged in research long-term. Such multi-year research experiences result in a number of benefits for undergraduates (Thiry et al., 2012; Adedokun et al., 2014), and in our lab this commitment builds a strong sense of shared ownership as teams develop a robust set of collective skills over time. This maximizes the usefulness of each student's contributions to their team as they learn and grow, with each team in turn making a sustained contribution to publishable research. It also creates efficiencies for research mentors, allowing them to focus in particular on mentoring the team leaders, who are able to work as young colleagues in advancing the mentor's lines of research.

## Enhancing Students' Sense of Belonging

Our approach to mentoring undergraduates is supported by research on team-based learning and leadership (Hackman, 2002) and work underscoring the importance of creating a sense of belonging among undergraduate students, especially those from traditionally underrepresented groups (Walton and Cohen, 2007). When students first make the college transition, it's natural for them to question the extent to which they belong at their new institution, but first-generation and minority students often fail to recognize that *all students* feel the same way. Helping these students appreciate this early on can transform subsequent challenges into evidence that they have things in common with other students and are a valued member of the community (Walton and Brady, 2017). For this reason, we intentionally engage students early in their college careers, often in their first semester. For example, with funding from the Sherman Fairchild Foundation and, previously, the National Science Foundation (NSF), we recruit first-generation students to our lab in their first month of college. This effort is designed to help increase first-generation students' levels of achievement and persistence in STEM and related fields, building on findings that suggest minority students are more likely to persist and achieve positive academic outcomes if they engage in undergraduate research (e.g., Jones et al., 2010; Clayton-Pedersen et al., 2017).

Our first-semester recruits often stay with us throughout their 4 years at the college, setting them up to become team leaders in their senior year. This continuity of engagement creates a built-in community where all students know they belong, and it also enables students to see a series of research studies through to fruition. This latter quality is essential to the mentor's program of research—it enables undergraduates to build the individual and collective skills needed to be sufficiently expert so they can conduct high-quality research efficiently and contribute collaboratively to the writing of a publishable manuscript.

## Building Bridges

We have successfully leveraged our team-based approach to build bridges to high school students as well as community college students. High school students (including those from underrepresented backgrounds) can be incorporated into summer research teams at the assistant level and paired with one or two college students who provide near-peer mentoring. The challenge is to identify students who are sufficiently prepared to benefit from the summer research experience. We have accomplished this by partnering with a small number of high schools and having our undergraduates design and deliver exciting research-based lessons at these schools. This generates a pool of potential applicants, and those identified as ready for a summer research experience are invited to apply. Nearly the same approach can be used with community college students, with instructors at partner schools identifying a pool of candidates prepared to do summer research at the team associate level. Bringing community college students in as associates (rather than assistants) is essential to foster their sense of belonging (i.e., avoiding the combination of their being fellow undergraduates but nevertheless outsiders *and* low status).

## Teaching Collaborative Writing

To produce publishable manuscripts in a team-based setting, we find it most productive to share the writing process with the most senior members of our research teams. Their team-based growth over at least one full year (plus coursework in statistics and research methodology) has prepared them for the technical demands of writing a publishable manuscript, and we make this trajectory clear to all students at the time they join our lab (see Table 2). We then adopt a best practices approach to the writing process (Silvia, 2007; Detweiler-Bedell and Detweiler-Bedell, 2013):

- Successful writing comes from breaking the process up into small, manageable steps. Outline the paper before beginning to write, then set regular deadlines for each step in the writing process.
- Collaborative writing is an iterative process, characterized by periods of solitary writing, peer editing, exchange of ideas, and team-based discussion.
- Because the skills necessary to write papers in psychology take time to develop, avoid giving ownership of sections to particular individuals. Conduct round-robin editing, where team members trade sections and take turns adding to and editing content.

**Table 2:** Policy agreement signed by new lab members.

	**To foster a successful, productive, and ethical research experience, we use the following set of policies for all student members of our research teams:**
Ethical obligations	All team members are required to follow the American Psychological Association's (APA) guidelines pertaining to the participation of human subjects in psychological research. This includes, but is not limited to, using only research materials that have been approved by the appropriate institutional review board committee, securing informed and free consent from all study participants, and keeping participants' identities and data strictly confidential. In addition, team members agree to have all research materials and procedures approved by one of the faculty advisors prior to implementation. Finally, team members agree to follow APA guidelines in properly citing the work of others. Academic integrity is an essential part of the research process. Plagiarism or the deliberate misrepresentation of any information or data is unacceptable.
Authorship expectations	On poster presentations, all active team members and the faculty advisors will be listed as co-authors. Other scholarly works (i.e., journal articles, book chapters) generally will be co-authored by the faculty advisors and the team leader(s), whose team-based growth over at least one full year on top of their coursework in statistics and research methodology has prepared them for the technical demands of writing a publishable manuscript. In some instances, at the discretion of the faculty advisors, a team associate also may be included as a co-author of these works. Order of authorship will be determined by level of involvement in conducting the research and writing the manuscript. Research associates and assistants who participated in aspects of the research project (e.g., data collection) but were not involved in its write-up will be acknowledged (thanked) in these works.
Team responsibilities	Team members are expected to carry out all of their obligations as described above. These obligations include regularly attending collaborative research meetings and activities as well as consistently carrying out individual work assigned by the team. Students choosing not to remain in the lab can step down at any time. Students not upholding their obligations or failing to abide by these policies will be asked to step down from their positions, and replacements will be made by the faculty advisors.

This egalitarian approach to writing consolidates the team's vision, collective feeling of ownership, and sense of togetherness. Regularly sharing progress gives meaning and longevity to the team's project and final written product.

## Securing Funding

Publishable research often starts with and is funded by a well-conceived grant proposal. Treating grant proposals as if they were themselves a publication, and involving undergraduates in writing them, can strengthen proposals and speed up proposal writing (especially at smaller institutions where grant writing is otherwise a lower priority relative to teaching). Moreover, adopting and describing a structured, team-based approach to undergraduate research provides evidence of the resources necessary to bring high-quality faculty-student collaborative research to fruition. It also gives proposals a distinct advantage in terms of their broader impact. Organizations such as NSF require grant proposals to demonstrate not only the publishable, intellectual merit of a project, but also the broader impact of the work (National Science Foundation, 2016). In our experience, the team-based model we've described is compelling to reviewers in terms of its ability to provide transformative research experiences to a broad array of undergraduates. This strength can be leveraged regardless of institution type, and it can set the stage for impactful collaborations between different types of institutions, as described earlier. Our experience on both sides of the table—securing grants and reviewing grant proposals—attests to how compelling it can be to do research in a way that demonstrably includes and impacts students from diverse backgrounds.

## Conclusion

Whether you are a faculty member or a graduate student working with undergraduates, a systematic approach to mentoring undergraduates lays the foundation for the creation of publishable research. Grounded in best practices, our structured, team-based model provides high quality mentorship, and a strong sense of belonging for students, while mitigating the challenges of securing funding and producing high-quality publishable research with undergraduates (for alternative approaches, see Miller et al., 2008). This type of approach is flexible and can be tailored to a wide range of settings from PUIs to community colleges, and from summer programs that bridge different types of institutions to teams of undergraduates led by graduate students at a research university. Most importantly, it works, as demonstrated by the success we have had in providing this systematic mentoring to 9–15 students per year and in sending half of these students to doctoral and other graduate programs in psychology or a related field (Detweiler-Bedell et al., 2010), together with our ability to obtain individual and institution-wide extramural funding and to publish with students at a consistent rate (about every other year) in the context of a small liberal arts college.

## Author Contributions

BD-B and JD-B contributed equally to writing this opinion piece. Each author drafted different parts of the opinion piece, edited and added to the entire manuscript, and worked together to finalize and submit. BD-B is listed as the first author alphabetically and JD-B is the corresponding author.

### Conflict of Interest Statement

The authors declare that the research was conducted in the absence of any commercial or financial relationships that could be construed as a potential conflict of interest.
